# Topologically Optimized Nano-Positioning Stage Integrating with a Capacitive Comb Sensor

**DOI:** 10.3390/s17020257

**Published:** 2017-01-28

**Authors:** Tao Chen, Yaqiong Wang, Huicong Liu, Zhan Yang, Pengbo Wang, Lining Sun

**Affiliations:** Jiangsu Provincial Key Laboratory of Advanced Robotics & Collaborative Innovation Center of Suzhou Nano Science and Technology, Soochow University, Suzhou 215123, China; chent@suda.edu.cn (T.C.); 20154229003@stu.suda.edu.cn (Y.W.); pbwang@suda.edu.cn (P.W.); lnsun@hit.edu.cn (L.S.)

**Keywords:** nano-positioning stage, multi-objective topological optimization, comb sensor

## Abstract

Nano-positioning technology has been widely used in many fields, such as microelectronics, optical engineering, and micro manufacturing. This paper presents a one-dimensional (1D) nano-positioning system, adopting a piezoelectric ceramic (PZT) actuator and a multi-objective topological optimal structure. The combination of a nano-positioning stage and a feedback capacitive comb sensor has been achieved. In order to obtain better performance, a wedge-shaped structure is used to apply the precise pre-tension for the piezoelectric ceramics. Through finite element analysis and experimental verification, better static performance and smaller kinetic coupling are achieved. The output displacement of the system achieves a long-stroke of up to 14.7 μm and high-resolution of less than 3 nm. It provides a flexible and efficient way in the design and optimization of the nano-positioning system.

## 1. Introduction

With continuous developments of the integrated circuit (IC) manufacturing process, ultra-precision machining, and bio-medical engineering, long-stroke and precise nano-positioning technology have been one of the hottest research areas in precision engineering [1,2]. Recent research has focused on the design and manufacturing of nano-positioning stages and high-precision sensors, as well as precise control methods [3,4,5,6]. Compared with traditional bulk mechanical stages, the nano-positioning stages have shorter product lifecycles and higher performance indicators to satisfy the development requirements of microelectronics [7]. The flexible and efficient design methodologies are urgent for meeting the high demands of nano-positioning stages.

Topology optimization, as a kind of conceptual design methodology, could be a choice of priority [8]. Different from the traditional optimizations of sizes and shapes [9], the topology optimization can achieve an optimal structural layout for different conditions without prior information. Meanwhile, the objective function of topology optimization, as design variables, can be designed to achieve the diversification of product design according to different requirements. Related to the initial design and post processing of the nano-positioning stage, the topology optimization can enhance and improve the stage performance significantly [10,11]. Ansola et al. [10] applied an evolutionary structural optimization (ESO) procedure for compliant mechanism design. They introduced the elastic strain energy of the mechanism within the mechanical advantage expressions used as objective functions, in order to improve convergence in the optimization. Chen et al. [11] applied the topology optimization in the size optimization of the stage and enhanced the static and dynamic characteristics significantly. In order to overcome the defects of traditional testing sensors, such as being oversized and low accuracy, high-precision nano-displacement sensors have emerged. Among these, the capacitive sensors have become the first choice of the testing devices, due to their high precision, good sensitivity, and high stability [12,13]. To overcome the drawbacks of limited range of capacitive testing, the capacitive sensors are generally made into offset constant-tooth comb-finger structures to satisfy the range and accuracy demands of nano-positioning [14,15].

The proposed nanopositioners above all have a good performance providing tens of micrometers range of displacement with nanometer resolution and a high bandwidth. However, the structures of the nanopositioners are more complicated, which may cause the fabrication difficulties and high costs. In addition, regarding the comb drive capacitive sensors, researchers are tending to design nanopositioners with a monolithic structure to form a closed-loop control. Here in this paper, we have adopted the topology optimization approach to achieve a wide displacement range and a fine resolution of the nano-positioning stage. In addition, the comb drive capacitive sensors and the nanopositiong are separately designed and connected with each other using a simple connection. The overall structure is relatively simple, and manufacturing cost is very low. Such an idea has proved to be a bold attempt and has also achieved a good result. The goal of this project is to develop an integrated model of micro displacement precision positioning system. The requirements of this system can achieve 10 μm travel, repeat positioning accuracy with 10 nm, and displacement resolution with 5 nm. This paper presents the displacement positioning system of a 1D stage at the nanometre scale by using a microelectromechanical system (MEMS)-based displacement sensor. The 1D nano-positioning stage is optimized by multi-objective topology optimization. A wedge pretension has been applied to verify the accuracy of the stage. An on-chip sensor of comb-finger capacitor is micro-fabricated with a high displacement sensing resolution and is constructed with the stage as a testing device to measure the displacement change of the stage. Experimental results of the travel distance and motion precision of the displacement-controlled manipulation are presented. The performance of the topology stage is superior to the traditional one. Through the combination with the comb sensor, the nano-positioning system can realize the installation of miniaturization and integration, competent for the requirement of precision engineering.

## 2. Nano-Positioning Stage

### 2.1. Device Configuration

**Figure 1:**
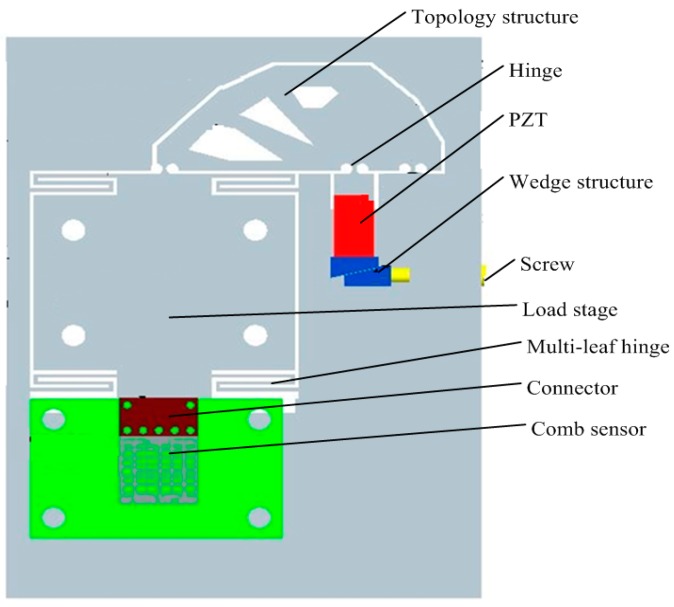
Schematic drawing of the nano-positioning stage device.

The schematic drawing of the proposed nano-positioning system is shown in Figure 1. It is comprised of a piezoelectric ceramic (PZT) actuator, a lever amplification mechanism, a load stage, and a capacitive comb sensor. The fabrication technique of the stage is wire-electrode cutting. Four multi-leaf springs are designed and machined according to the stiffness requirement. The lever connects the load stage and the PZT actuator by hinges. The topology structure on the lever includes three notches. A preloading screw provides a preload compressive force on the wedge structure for maintaining the feeding accuracy of the PZT actuator. The working principle of the nano-positioning stage is to drive flexible hinges to achieve nanometer motion through the inverse piezoelectric effect of PZT. The whole stage is made of duralumin (Al-Mg alloy, LY11). The size of the comb sensor is 10 mm × 10 mm × 1 mm, including 6 × 70 pairs of differential combs, and its maximum travel distance can achieve 15 μm. The size of the stage, decided by the PZT ceramics, is currently 60 mm × 80 mm × 20 mm, and it can be further reduced to 30 mm × 30 mm × 10 mm. The design objective is to obtain a maximum output displacement of up to 10 μm as well as positioning accuracy of smaller than 5 nm, with high stiffness, good dynamic property and system stability.

### 2.2. Modeling and Optimization

In traditional design, both the kinematics and structure must be considered, while the structure design is normally ignored due to the optimal rule [16]. We use mutual energy concept [17] to describe the requirements as follows:

(1) $$\begin{array}{cc} L^{k}(u^{l})=L(t^{k},u^{l})=\int_{\Gamma_{tk}}t(k)u^{l}d\Gamma & u^{l} \end{array}\in V,$$

(2) $$a(u^{k},v^{l})=\int_{D}\varepsilon {\left(v^{l}\right)}^{T}E\varepsilon (u^{k})dD,$$

(3) $$\begin{array}{cc} a(u^{k},v^{l})=L^{k}(v^{l}) & u^{k}\in V,\forall v^{l} \end{array}\in V,$$

where *t*(*k*) is the force at the point *k*, and *u^l^* is the displacement at the point *l*. Γ is the point sustaining force, and D is the design area. *a*(*u*,*v*) is the energy of virtual work and *v* is the virtual displacement. The energy of external force is calculated by using Label (1), and the energy of virtual work is calculated by using Label (2).

**Figure 2:**
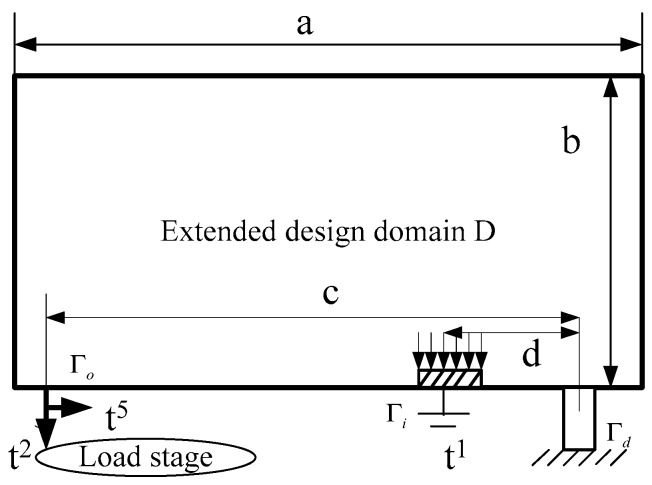
Design area of the topology optimization.

The requirement of kinematics, in order to maximize mutual mean compliance *L^q^*(*u^p^*), means that sufficient amount of deformation can be produced in the specified direction “*p*” under a specified input “*q*”. Regarding the requirement of structure, the stiffness of some parts or portions “*n*” must be maximized to achieve high performance, and to minimize compliance *L^n^*(*u^n^*). In this paper, topology optimization has been conducted in the lever amplifier design of the stage, and the design area must be extended to avoid concave problems [18]. As can be seen in Figure 2, a and b are the length and width of extended design domain, respectively; c and d are the distances from the pivot to the output and input hinges, respectively. The input displacement *t*^1^ is applied by the PZT actuator, while the output displacement *t*^2^ is caused by the counter force of the load stage. In order to reduce the cross coupling [19,20] in the vertical direction and facilitate the amplification expansion of the stage, the force *t*^5^ is added perpendicular to *t*^2^, in order to decrease the movement in the vertical direction and ensure the continuous goal direction.

On the basis of the virtual work principle, it is set as *t*^3^ = *t*^1^ and *t*^4^ = −*t*^2^. Thus, the design can be described as a multi-objective problem, consisting of four objective functions. In the case, the mutual mean compliance *L*(*t*^2^,*u*^1^) must be maximized; the mean compliances *L*(*t*^3^,*u*^1^) and *L*(*t*^4^,*u*^2^) must be minimized; and the mutual mean compliance *L*(*t*^5^,*u*^1^) must be minimized. To find the incorporation optimal solution, the multi-objective function is formulated as:

(4) $$\max f_{1}=\frac{L(t^{2},u^{1})}{{\left(w_{s}L{\left(t^{3},u^{1}\right)}^{2}+(1-w_{s})L{\left(t^{4},u^{2}\right)}^{2}+w_{M}L{\left(t^{5},u^{1}\right)}^{2}\right)}^{\frac{1}{2}}}.$$

To be more general:

(5) $$\begin{array}{l} \max f_{2}=W\log(L^{2}(u^{1}))\\ -\frac{1}{2}(1-W)\log(w_{s}L^{3}{\left(u^{3}\right)}^{2}+(1-w_{s})L^{4}{\left(u^{4}\right)}^{2}+w_{M}L^{5}{\left(u^{1}\right)}^{2}) \end{array},$$

where *W*, *w_s_* and *w_M_* are the weight coefficients. *W* determines the weight between mutual compliance *L*(*t*^2^,*u*^1^) and mean compliance *L*(*t*^3^,*u*^1^), *L*(*t*^4^,*u*^2^) as well as *L*(*t*^5^,*u*^2^). *w_s_* determines the weight between mean compliance *L*(*t*^3^,*u*^1^) and mean compliance *L*(*t*^4^,*u*^2^). *w_M_* is the weight coefficient for the relative deformation constraint.

To get a specified optimization structure, the effect of weighting coefficients *W*, *w_s_*, and *w_M_* on the optimal configurations are investigated. The relationship between the optimal configuration and weight coefficient *W* is shown in Table 1. It is clear that, as *W* increases, the mutual mean compliance *L*(*t*^3^,*u*^1^) and mean compliances *L*(*t*^4^,*u*^2^) increase. That is, the flexibility increases while the stiffness of the input and output decrease. The similar trend of transformations can be obtained as the change of *w_s_* and *w_M_*. Then, the suitable weight coefficients can be selected to specify the configuration.

**Figure 3:**
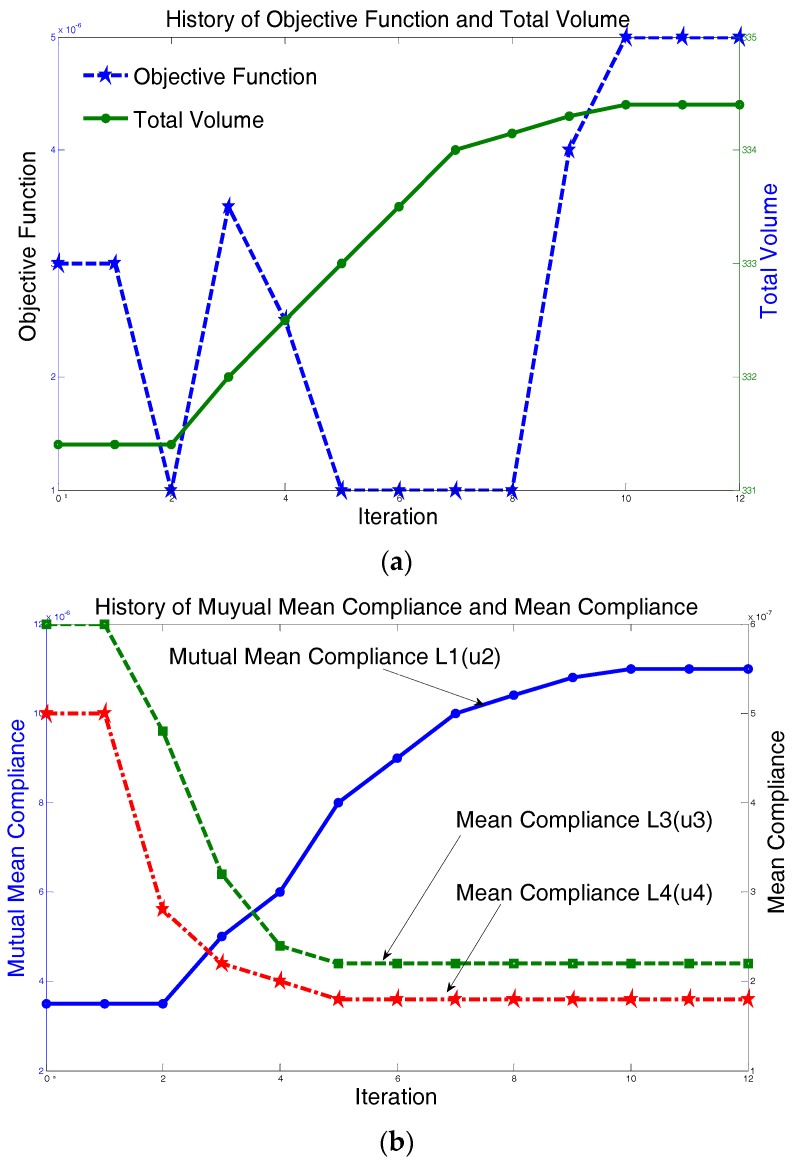
(**a**) History of the objective function and total volume; and (**b**) History of mutual compliance and self-compliances.

As the parameters of the multi-objective function are determined, the convergence history of the topology optimization process can be drawn through the finite element method. Figure 3a illustrates the convergence of the objective function (in blue) and the relationship between the objective function and total volume (in green). It is noted from Figure 3b that the mutual compliance *L*^2^(*u*^1^) increases as the iteration continues, while the compliances *L*^3^(*u*^3^) and *L*^4^(*u*^4^) decrease.

Finally, the preliminary configuration of the nano-positioning stage is obtained. However, due to the chessboard problem and partial polarization [21], the preliminary configuration produces some “transitional element”. Meanwhile, due to the complex and ambiguous distribution, it is difficult to pick up the element from the topology configuration. Thus, the image-based design method [22] is employed to draw the topology structure. At first, the characteristic function, i.e., pixels, is defined instead of the element density. Then, the distribution of topology optimal configuration for each element is represented by the integer 0 to 27 scale value. By setting an appropriate threshold, the integer scale values are converged, through the method of density penalty [23], to “0” and “1”. The scale values that are larger than the threshold form the real structure, and the ones that are smaller than the threshold form the hole. Sometimes, some fitting curves are necessary in order to make the topology configuration behave well in terms of manufacturability.

### 2.3. Simulation Analysis

In order to verify the superiority of the multi-objective topology optimization, a traditional MPT-1JR micro-stage (designed by Jiangsu Huibo Robotics Technology Co., Ltd., Suzhou, China) with the same size optimization has been applied as well. Both the static and dynamic characteristics are compared.

**Figure 4:**
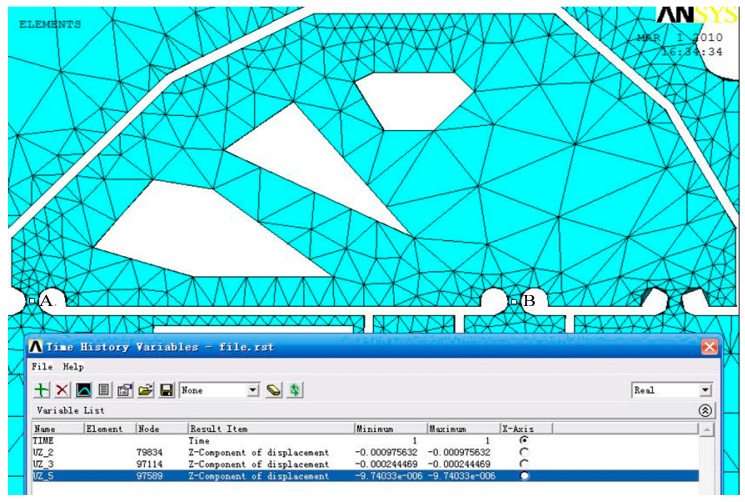
Static analysis of the output displacements at points (**A**) and (**B**) of the hinge with topology design.

**Figure 5:**
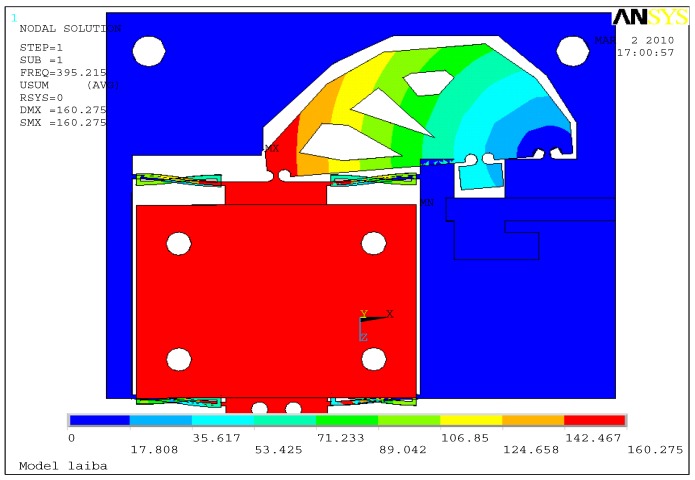
The first resonant mode shape of the topology optimum design.

(1) Static analysis: On the basis of finite element modelling, static analysis is conducted and the input and output displacements of the hinges at points B and A are studied as shown in Figure 4. The input and output displacements of the points in the core of a circular hinges stand for the mean displacements of the nano-positioning stage. Through the multi-objective topology optimization, the maximum output displacement, amplification ratio, as well as the displacement damage in the PZT are calculated and summarized in Table 2. Compared with the traditional size optimization, it is found that the maximum output displacement and the amplify ratio of the topology design increase, while the input remains approximately the same level. As the goal displacement feed is obtained, the topology design can reduce the damage in PZT because of the reduction of reaction force.
  Besides the kinematics requirement, the structure requirement is considered in the multi-objective topology. The two mean compliances *L*(*t*^3^,*u*^1^) and *L*(*t*^4^,*u*^2^) at the input and output points remain sufficiently stiff. Based on the ANSYS simulation results as shown in Table 3, the reduction of the stress values can be clearly observed, that is, the stiffness of the input and output gain the increase. Meanwhile, the stiffness changes of the output and pivot are similar due to the mechanism principles. The vertical displacement of the goal direction is tested. It is found that a small coupling ratio is suitable for the goal requirement and propitious to expand the multi-dimensional stages with reduced cross coupling.
(2) Dynamic characteristics: The stability is dominated by the first resonance mode of the body structure, which is normally a bending mode. In order to obtain high stability, the nano-positioning stage must be designed to raise the frequency of the first resonance mode as high as possible. The mode analysis is performed as shown in Figure 5. The resonance frequency is found to increase from 391.7 to 470.3 Hz through the multi-objective topology optimization. The resonance response of the nano-positioning stage is studied. When the PZT starts or stops operating, because of the characteristics of the PZT ceramic itself, such as creep or hysteresis, the system will result in some frequency disturbance. For a traditional MPT-1JR micro-stage, the frequency response in the PZT ceramics is higher than the one in micro-stage, and oscillation will come up to make the entire system unstable. Through topology optimization, the frequency response can be effectively improved to avoid the emergence of such disturbance.

## 3. Capacitive Comb Sensor

### 3.1. Structure Configuration

**Figure 6:**
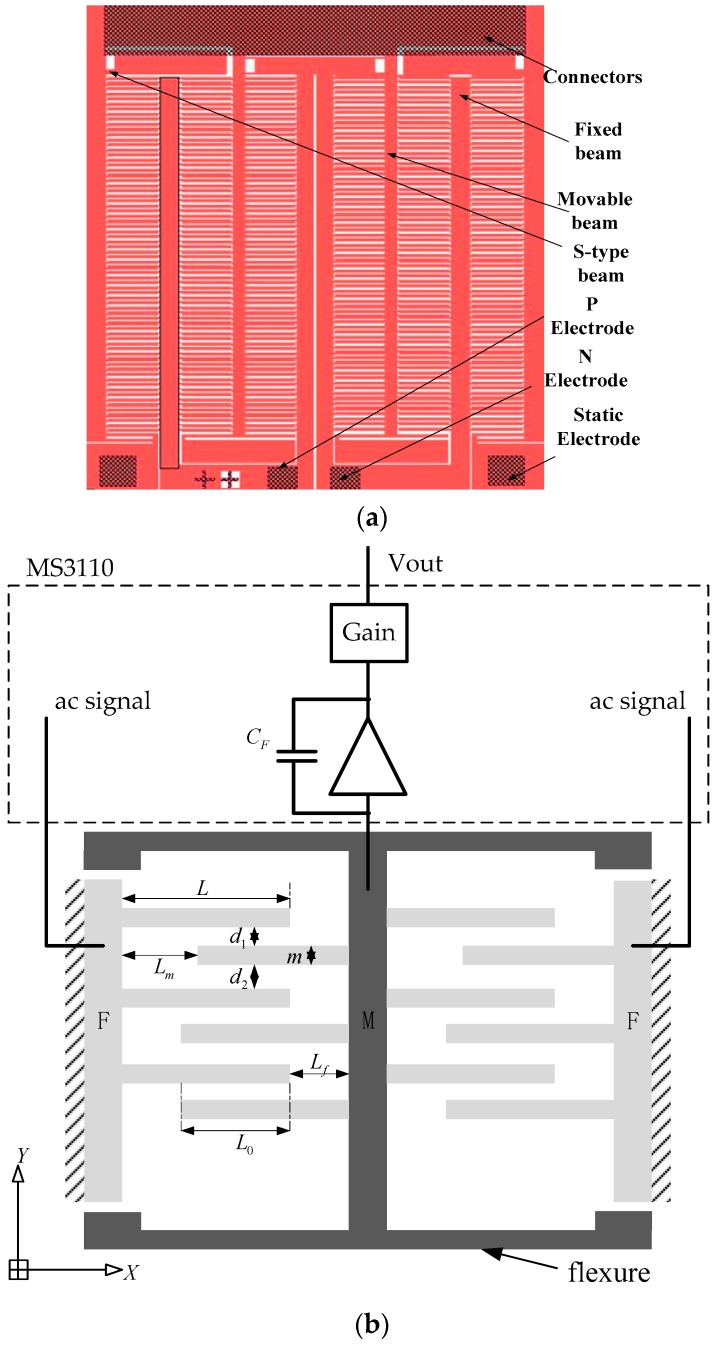
Schematic drawing of (**a**) the comb sensor and (**b**) the offset constant-tooth structure with readout circuitry

To overcome the hysteresis and nonlinearity defects of the PZT ceramic and improve the positioning accuracy of the overall system, it is necessary to integrate a displacement sensor with high precision and miniaturization to form a feedback system. As shown in Figure 6a, the capacitive comb sensor consists of fixed comb electrodes, movable comb electrodes, S-type beams and flexible cantilevers. The offset constant-tooth structure with the actuation displacement in the *y-*direction in Figure 6b has been applied in the comb sensor to compensate the nonlinearity and hysteresis effect of piezoelectric ceramic and improve the positioning accuracy. In the figure, *F* is the fixed beam, *M* is the movable beam, *t* is the thickness of comb electrodes, m is the width of electrodes, d is the gap between *F* and *M* electrodes, *L*_0_ is the overlap distance of the electrodes, *L_m_* and *L_f_* are distances from the top to end of the electrodes, and *L* is the length of each electrode. The specific interior parameters of the comb, including the comb electrodes and flexible beam, are determined by the stroke range and resolution of the nano-positioning system as shown in Table 4. The S-type beam is used to reduce the stiffness of the beam and improve the flexibility.

### 3.2. Manufacturing Process

**Figure 7:**
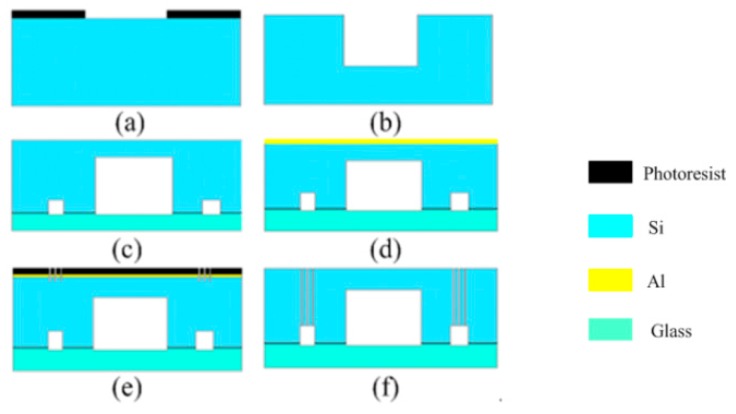
Process sequence of the micro comb sensor. (**a**) Oxidation and lithography; (**b**) Dry etching; (**c**) Bonding of silicon and glass; (**d**) Sputtering of aluminum; (**e**) Lithography of structure; (**f**) Etched using DRIE.

The capacitive comb sensor is microfabricated by a series of MEMS processes as shown in Figure 7.

(a) It is started from a 4-inch, N-type, (100) orientation, double polished silicon (Si) wafer. The back release windows are patterned on the backside of the wafer; (b) a backside deep reactive ion etching (DRIE) process is performed to form a depth of 250 μm; (c) then, a glass wafer is bonded onto the Si wafer to form the support base; (d) the aluminum layer of 1-μm-thick is formed by vacuum evaporation; (e) the aluminum wires and bonding pads are formed by etching processes and then the structure of sensor is patterned on the wafer; and, (f) finally, the device layer including the comb fingers and the flexure beams are etched using DRIE.

**Figure 8:**
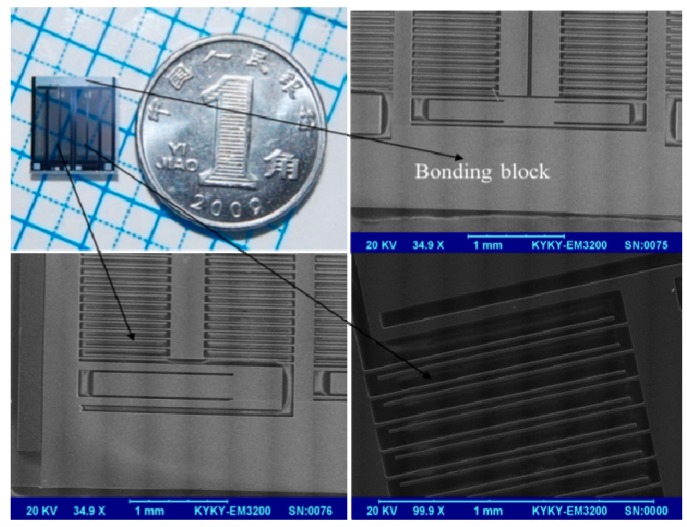
Photograph and enlarged views of the comb sensor by SEM.

With the combination of surface and bulk micromachining processes, the comb sensor demonstrates good detective performance of precision scale and is easy to integrate with the nano-positioning stage. The photo and SEM images of the sensor are shown in Figure 8.

## 4. Experiments

**Figure 9:**
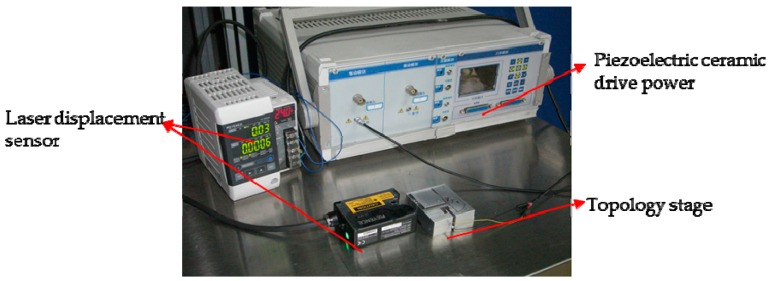
Testing system of the nano-positioning stage with optimized topology structure.

Experiments have been conducted to verify the simulation results. The testing system as shown in Figure 9 includes a Keyence LK-G CCD Laser Displacement Sensor (Keyence, Osaka, Japan), a PTBS200 piezoelectric PZT ceramic (Boshi, Harbin, China), and the fabricated nano-positioning stage. A dual-frequency laser interferometer ZLM700 (JENAer, Jena, Germany) is used to test the resolution of the stage. The stage driven by the piezoelectric ceramic PTBS200/5 × 5/5 is manufactured with a duralumin alloy (LY11), which has a material density of 2.7 g/mm^3^, Young’s modulus 89 GPa, Poisson ratio of 0.33 and yielding strength of 505 MPa. The size of the stage is 80 mm × 60 mm × 20 mm.

**Figure 10:**
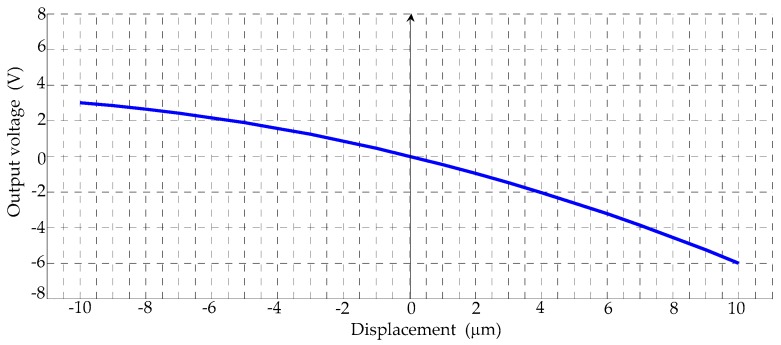
Input and output calibration of the comb sensor.

**Figure 11:**
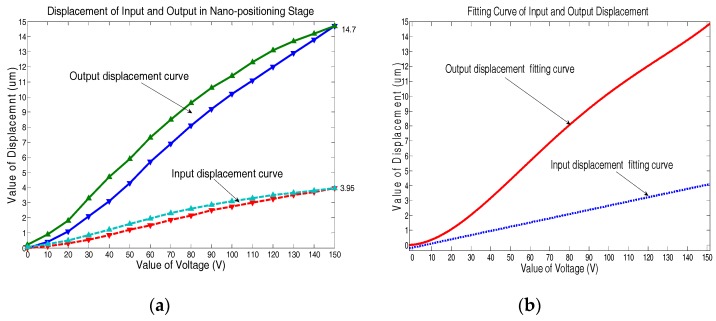
(**a**) The input and output displacements of the stage; (**b**) high-order displacement fitting curves of the input and output displacements.

The calibration curve of the comb sensor is provided in Figure 10 according to the relationship between physical change and electrode spacing change. It can be seen that the measurement range of the comb sensor should between −5 μm and 5 μm for a better linearity and the linearity error of the sensor is 3%. The hysteresis curves of the input and output displacements of the stage at points B and A are shown in Figure 11a. As can be seen, the output displacement is up to 14.7 μm at 150 V. On the basis of the output displacement curve, the resolution of the nano-positioning stage is results in being less than 3 nm for an input voltage increment of 1 mV by the Laser Displacement Sensor. To compare the change of the output characteristics and amplifier ratio of the topology optimized stage, a high-order curve fitting has been done as shown in Figure 11b. Referring to Labels (7) and (8), the amplification ratio is about 3.99, which is essentially consistent with the finite element simulation result. It shows that topology optimization can improve the static characteristics of the nano-positioning stage.

**Figure 12:**
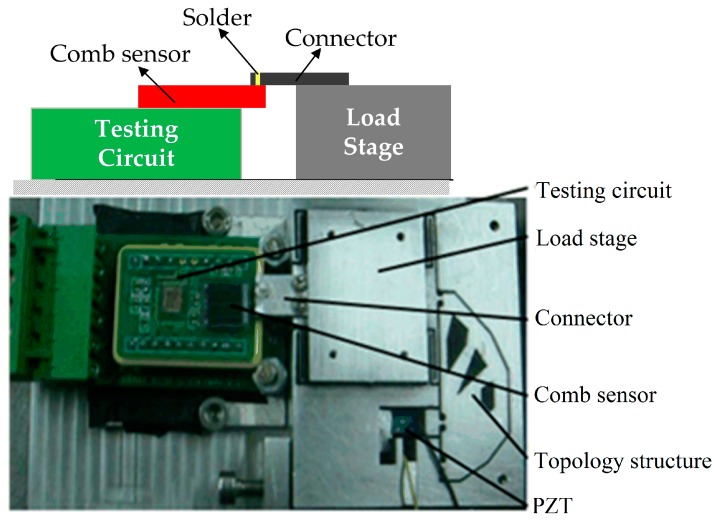
The nano-positioning system integrating with the capacitive comb sensor.

For an applied voltage of 150 V, the displacements along the *x*-direction is 14.7 μm, while along the *y*-direction, it is 0.1 μm. Thus, the cross coupling ratio is 0.6%, which is mainly due to the manufacturing issues, and the actual parts of the topology in the corner and the inflection point have maching errors. In order to control the precision of the nano-positioning stage, the output displacement of the stage is calibrated by the integrated comb sensor before the next closed loop. Figure 12 shows that the comb sensor is suitable for integration and miniaturization in the design of nano-positioning systems.

**Figure 13:**
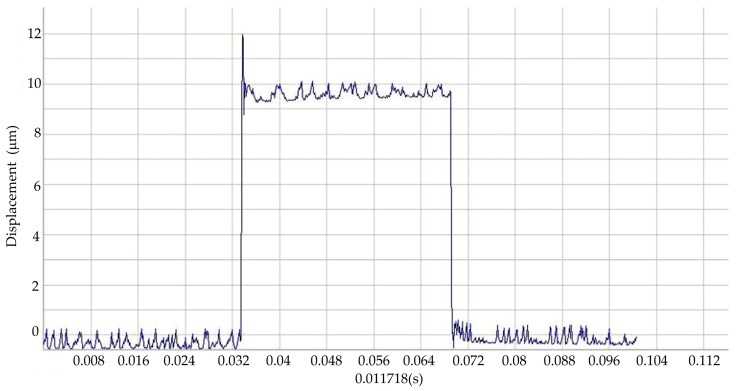
The dynamic testing of the nano-positioning system.

To test the dynamic characteristics of the system, the PZT power device provides the full value of the step voltage. Then, a laser interferometer is used to measure the corresponding time, as shown in Figure 13. We can see that the response time of rising edge is 2 ms. Therefore, the natural frequency of the system is about 500 Hz.

In order to verify the repetitive positioning accuracy and the stability time of system designed, the displacement response test and the repeat positioning tests of fixed points are carried out, respectively. With the control system commands, the microstage is located at the 10 μm position. An oscilloscope is used to test the response curve of control. The results show that the whole control process of rising is stable. There is no disturbance and overshoot.

With the control system, the microstage is positioned at the centre position (about 7.4 μm), and 10 sets of data are tested. We can see that the repeat positioning accuracy is 10 nm as shown in Table 5. It fully meets the design requirements.

## 5. Conclusions

This paper presents a novel nano-positioning system with high-precision, large-stroke and high-integration. The multi-object topology optimization is applied to increase the amplification ratio, reduce the stiffness distribution and improve the frequency property of the nano-positioning stage. Based on the static test, the stiffness at the input and output points is increased and the coupling ratio is reduced. Through the reduction of the whole stiffness, resonant frequency of the nano-positioning system is increased, avoiding the resonance induced oscillation and improving the stability of the system. Combined with the offset constant-tooth comb sensor, the nano-positioning system realizes the installation of integration and miniaturization.

## Figures and Tables

**Table 1:** Changes of the mutual mean compliance and mean compliance.

Weight *W*	*L^2^(*u*^1^)	*L^3^(*u*^3^)	*L^4^(*u*^4^)
0.3	130	1.0	0.5
0.5	150	1.3	1.0
0.7	190	2.0	1.5
0.9	500	50	12

*L^2^(*u*^1^): Mutual mean compliance; *L^3^(*u*^3^), *L^4^(*u*^4^): input and output mean compliance.

**Table 2:** Comparison of the static properties of the topology optimized stage.

	Traditional Optimization	Topology Optimization	Comparison (%)
Output(μm/N)	0.907	0.9756	5.4
Input(μm/N)	0.246	0.2445	2.0
Amplify Ratio	3.68	3.99	8.4
Damage in PZT(nm/N)	11.03	10.46	−5.17

**Table 3:** Comparison of the static properties of the topology optimized stage.

(MPa/N)	Output	Input	Pivot
Original	0.206	0.11	0.299
Optimum	0.16	0.037	0.23
Reduction (%)	↓22.3	↓66.4	↓23

**Table 4:** Design parameters of the comb sensor.

Parameters	Range
Thickness t	200 μm
Width m	15 μm
Gap d_1_	15 μm
Gap d_2_	50 μm
Length Ls	10–15 μm
Overlap L_0_	880 μm
Voltage V	−8 V–8 V
Comb number n	420

**Table 5:** Position repeatability of the system (unit: μm).

P1	P2	P3	P4	P5	P6	P7	P8	P9	P10
7.402	7.405	7.406	7.398	7.406	7.402	7.400	7.403	7.405	7.401
